# Retrospective clinic and urodynamic study in the Neurogenic Bladder Dysfunction caused by Human T Cell Lymphotrophic Virus Type 1 Associated Myelopathy / Tropical spastic paraparesis (HAM/TSP)

**DOI:** 10.1186/1742-4690-12-S1-P75

**Published:** 2015-08-28

**Authors:** O Troisgros, F Naghibzadeh Darbon, JL Barnay, P Olive, F Komminoth, P René-Corail

**Affiliations:** 1 Service MPR-CHU Fort De France

## Introduction

HTLV-I –associated tropical spastic paraparesis (TSP) and HTLV-I associated myelopathy (HAM) is an endemic disease in Carribean Island. Bladder-sphincter dysfunctions are almost present. Functional disablement is major. Few studies are devoted to urinary disablement, responsible for urologic complications, that gives one of the elements of the severity prognosis of the disease. The objectives of the study are first to describe clinic and urodynamic characteristics of voiding disorders in Martiniquan population, secondly evaluate if there is a relationship between motor and urinary handicap, and thirdly evaluate prognosis factors of urologic complications.

## Methods

Retrospective study of 60 patients suffering from HAM/TSP. Clinical, urodynamic datas, scale of urinary and motor handicap (MHU and Osame Score) were collected. For qualitative variables, the distribution of the frequencies associated is calculated. For quantitative variables, means are calculated. Chi2 test was used to compare percentages, means comparison test to compare qualitative and quantitative datas.

## Results

Irritative symptoms were the most frequent (75%) whatever type of detrusor activity. Detrusor over activity was the most frequent disorder (75.61%). Bladder compliance was normal in half percent of the cases. Urethral activity was increased in 47% of the cases. Detrusor sphincter dysynergia was found in 78% of the cases, post-void residual in 58% of cases. 65% of the patients present at least one urologic complication (morphologic and/or infectious) but there was no correlation with motor enablement (p=0.3097), neither urodynamic study (p=0.432 for detrusor overactivity, p = 0.107 for detrusor underactivity, p=0.058 for high urethral activity, p= 0.893 for detrusor sphincter dysynergia, p=0.850 for post-void residual volume), neither with evolution duration of HAM/TSP (p=0.348). MHU score was not in correlation with Osame score (p=0.09).

## Conclusion

Urologic symptoms are similar to the literature and are not always in relationship with urodynamic study: so a systematic urodynamic study is necessary to evaluate HAM/TSP neurogenic bladder. There is no relationship between urologic and motor handicap. No clinic or urodynamic criterias are predictive of urologic complications. These patients need a closer and regular follow up throughout life by a multidisciplinary team. A prospective study based on this prospective study is underway to precise the evolution of urologic disorders over time.

